# OsERF2 controls rice root growth and hormone responses through tuning expression of key genes involved in hormone signaling and sucrose metabolism

**DOI:** 10.1007/s11103-015-0416-9

**Published:** 2015-12-10

**Authors:** Guiqing Xiao, Hua Qin, Jiahao Zhou, Ruidang Quan, Xiangyang Lu, Rongfeng Huang, Haiwen Zhang

**Affiliations:** College of Bioscience and Biotechnology, Hunan Agricultural University, Changsha, 410128 People’s Republic of China; Biotechnology Research Institute, Chinese Academy of Agricultural Sciences, Beijing, 100081 People’s Republic of China

**Keywords:** OsERF2, Rice, Root growth, ABA, Ethylene, Sugar

## Abstract

Root determines plant distribution, development progresses, stress response, as well as crop qualities and yields, which is under the tight control of genetic programs and environmental stimuli. Ethylene responsive factor proteins (ERFs) play important roles in plant growth and development. Here, the regulatory function of *OsERF2* involved in root growth was investigated using the gain-function mutant of *OsERF2* (*nsf2857*) and the artificial microRNA-mediated silenced lines of *OsERF2* (*Ami*-*OsERF2*). *nsf2857* showed short primary roots compared with the wild type (WT), while the primary roots of *Ami*-*OsERF2* lines were longer than those of WT. Consistent with this phenotype, several auxin/cytokinin responsive genes involved in root growth were downregulated in *nsf2857*, but upregulated in *Ami*-*OsERF2*. Then, we found that *nsf2857* seedlings exhibited decreased ABA accumulation and sensitivity to ABA and reduced ethylene-mediated root inhibition, while those were the opposite in *Ami*-*ERF2* plants. Moreover, several key genes involved in ABA synthesis were downregulated in *nsf2857*, but unregulated in *Ami*-*ERF2* lines. In addition, OsERF2 affected the accumulation of sucrose and UDPG by mediating expression of key genes involved in sucrose metabolism. These results indicate that *OsERF2* is required for the control of root architecture and ABA- and ethylene-response by tuning expression of series genes involved in sugar metabolism and hormone signaling pathways.

**Electronic supplementary material:**

The online version of this article (doi:10.1007/s11103-015-0416-9) contains supplementary material, which is available to authorized users.

## Introduction

Root systems are central to the acquisition of water and nutrients, plant anchorage, seedling vigor, and responses to various stresses, which have pivotal effects on crop distribution, maximal productivity and yield stability, particularly in arid regions. Root architecture is under the tight control of genetic programs and environmental stimuli. It is well known that all phytohormones synergistically or antagonistically control root growth and development (Cuesta et al. 2013; Jung and McCouch 2013). In *Arabidopsis*, numerous crucial genes and intricate signaling pathways involved in root development have been well clarified, but many plant species also show their unique molecular and cellular regulatory mechanisms governing root development (Coudert et al. 2010).

Rice (*Oryza sativa*) is one of the most common crops worldwide. A series of mutants, genes, and genetic mechanisms governing root development have been characterized in rice, some of which are controlled by auxin and cytokinin (Mai et al. 2014). For examples, OsAUX1 controls auxin-mediated lateral root initiation through mediating polar auxin transport in rice (Yu et al. 2015). Mutation of *OsXXT1* causes abnormal root hair development with the reduction of XyG content and the tensile strength of the cell wall (Wang et al. 2014). Increasing auxin-related mutants including *crl1* (*crownless root1*), *arl1* (*adventitious rootless1*), *crl4/osgnom1*, and *oscand1*, exhibit abnormal root formation and growth due to the disorders of auxin biosynthesis, polar transport or signaling transduction in rice (Inukai et al. 2005; Liu et al. 2005, 2009; Wang et al. 2011). Moreover,auxin–cytokinin crosstalk plays curial roles in the control of root growth and development in rice. For examples, auxin-induced *CRL5* promotes crown root initiation through repressing cytokinin signaling (Kitomi et al. 2011). OsCKX4 and EL5 are essential for crown and lateral root development by mediating crosstalk between auxin and cytokinin pathways (Gao et al. 2014; Koiwai et al. 2007; Mochizuki et al. 2014). In addition, OsMPK3/6 and OsMKK4/5 are involved in the regulation of root architecture through tuning the interplay of auxin and cytokinin (Singh et al. 2015).

Sugar signals are central in determining plant growth and development by interacting with other signalling pathways (Lastdrager et al. 2014). In *Arabidopsis*, sucrose affects root architecture through regulating endogenous flavonols accumulation, which can suppress the effect of ABA on root growth (Nguyen et al. 2013). In rice, ABA mediates grain-filling rate by controlling the activities of key enzymes involved in sucrose-to-starch conversion in spikelet (Wang et al. 2015; Zhu et al. 2011). Moreover, several genes involved in sucrose synthesis and metabolism or transport including *OsSPS1*, *OsSUT1*, *OsSUT2*, *OsCYT*-*INV1*, and *OscFBP1* have been proved to be required for the control of rice growth and development (Eom et al. 2011; Hirose et al. 2010, 2014; Jia et al. 2008; Lee et al. 2008). For examples, mutation of *OsSUT2* affected sucrose and other sugars transport and caused growth retardation including tiller number, plant height, and root dry weight (Eom et al. 2011). Disruption of *OsCYT*-*INV1* impaired normal sucrose metabolism and root growth in rice (Jia et al. 2008).

Recent researches have proved that ethylene and its interaction with ABA play important roles in the control of rice growth and development. For examples, *MHZ4/ABA4*- and *MHZ5*-mediated ABA signaling is required for ethylene-induced inhibition of root growth (Ma et al. 2014; Yin et al. 2015). Moreover, *mhz6/Oseil1* and *mhz7/ein2* exhibit longer root, while overexpression of *MHZ6* and *MHZ7* represses rice root elongation (Ma et al. 2013; Yang et al. 2015). Importantly, the inducible expression of *OsERF2* (Os06g08340) by ethylene in roots was impaired in these mutants (Ma et al. 2013, 2014; Yang et al. 2015; Yin et al. 2015). In this paper, we found that OsERF2 was required for the regulation of primary root growth in rice. The gain-of-function mutation of *OsERF2* (*nsf2857*) shows short root, while artificial microRNA-*OsERF2* (*Ami*-*OsERF2*) lines exhibit longer root, this phenotype is consentient with that of *OsEIL1*. At the transcript level, OsERF2 negatively regulates expression of candidate genes including *ARF1*, *MKK4/5*, *MPK3/6* and *PIN1b/2/9*, which are involved in root growth and development through mediating auxin and cytokinin signaling. Furthermore, OsERF2 affects expression of key genes involved in ABA synthesis, which results in the enhanced accumulation of ABA in *Ami*-*OsERF2* lines associated with root growth hypersensitivity to ABA, while those are opposite in *nsf2857*. Moreover, OsERF2 influence accumulation of sucrose and UDPG through mediating expression of genes related to sucrose metabolism, including *INV* (Os01g0332100) and *OsCYT*-*INV1*, *OsSPS2*, *OsSUS3* and *OsSUS6*. Particularly, *OsCYT*-*INV1* is closely related to the accumulation of sucrose and root growth in rice (Jia et al. 2008). Thus, our results indicate that OsERF2 is required for the control of root architecture and ABA response by regulating expression of pivotal genes involved in root development, ABA synthesis and sucrose metabolism.

## Results

### OsERF2 negative regulates primary root growth

**Fig. 1:**
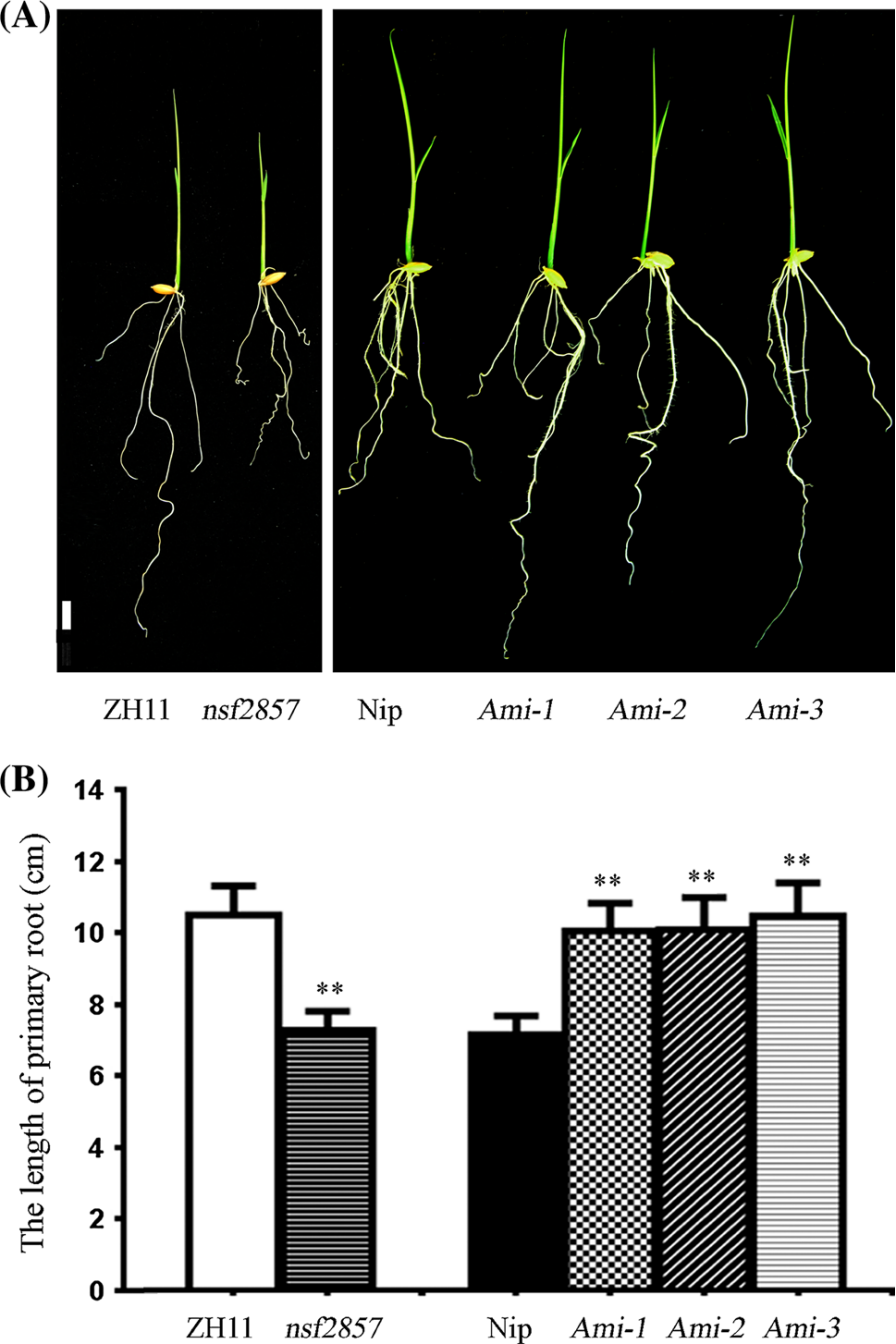
OsERF2 affects primary root growth. **a** Comparison of primary root length of 7-day-old seedlings between ZH11 and *nsf2857*, as well as Nip and three *Ami*-*OsERF2* lines with similar phenotypes, *bar* 1 cm. **b** Statistical analyses (*t* test) were performed with graphpad prism 6 for the primary root length of ZH11 and *nsf2857*, as well as Nip and *Ami*-*OsERF2*. The seedlings of each sample were more than 20. *Error bars* represent SD. **P < 0.01

Recent studies showed that the induction of *OsERF2* by ethylene was despaired in several ethylene-insensitive mutants *mhz4*-*7* with longer primary roots (Yang et al. 2015). To investigate whether *OsERF2* is involved in the regulation of root growth, we obtained the gain-of-function mutant of *OsERF2* (*nsf2857*) with T-DNA insertion in its promoter from the stock seeds in SHIP (http://ship.plantsignal.cn), and generated the artificial microRNA-mediated silencing lines of *OsERF2* (*Ami-ERF2*). After checked expression of *OsERF2* in different materials by qPCR (Figure S1), three *Ami*-*OsERF2* lines and *nsf2857* were chosen for further analyses. We first compared the root growth status of these materials under normal conditions. For 7-day seedlings, the whole root system of nsf2857 is inferior to that of ZH11. Particularly, the primary root system of *nsf2857* (about 6.97 cm) was significant shorter than that of ZH11 (about 10.49 cm). However, three *Ami*-*OsERF2* lines displayed longer primary roots (about 10.20 cm) compared with Nip (about 7.04 cm) (Fig. 1a, b). These results imply that *OsERF2* negatively controls primary root growth, and this phenotype is consistent with that of *OsEIL1* (Figure S2). Combined its inducible expression by key components of ethylene pathway such as OsEIN2 and OsEIL1, we speculate that OsERF2 might act as downstream regulator of ethylene signaling and is required for root growth and development.

### OsERF2 regulates expression of key genes related to root architecture

**Fig. 2:**
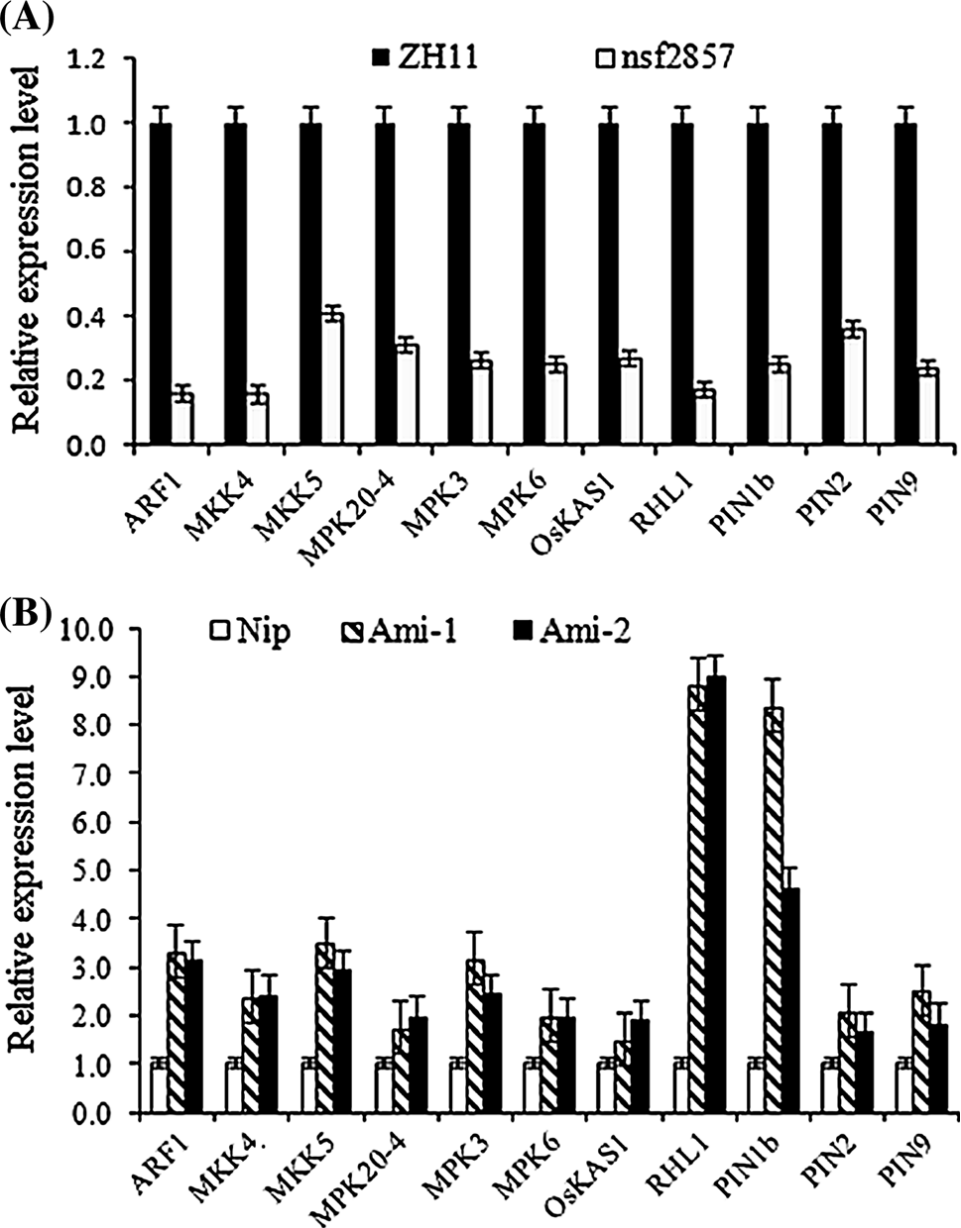
OsERF2 suppressing expression of key genes involved in root architecture. Relative expression level of candidate genes in *nsf2857* roots compared with ZH11 (**a**) and in *Ami*-*OsERF2* roots compared with Nip (**b**). Transcript level from ZH11 and Nip was set at 1, respectively. *OsACTIN1* was used as an internal control. *Data* represent the means of three repeats

To analyze the transcriptional regulation of *OsERF2*, we compared transcript levels of key genes involved in root growth between in *nsf2857*, *Ami*-*OsERF2* and WT seedlings. As shown in Fig. 2, expression of 11 candidate genes was downregulated in *nsf2857*, but upregulated in *Ami*-*OsERF2* lines. Among these genes, *ARF1*, *MKK4/5*, *MPK3/6* and *PIN1b/2/9* are involved in root growth through mediating auxin or cytokinin signaling. Moreover, *OsKASI* is required for rice root development by regulating fatty acid synthesis (Ding et al. 2015). *RHL1*, a homologs of *ArabidopsisRHL1* (ROOT HAIRLESS 1), is required for root hair initiation (Schneider et al. 1998). These results imply that OsERF2 negatively regulates primary root growth partly due to mediating expression of genes involved in auxin/cytokinin signaling or key functional genes closely related to root development.

### OsERF2 affects ABA accumulation and root growth response to ABA and ethylene

**Fig. 3:**
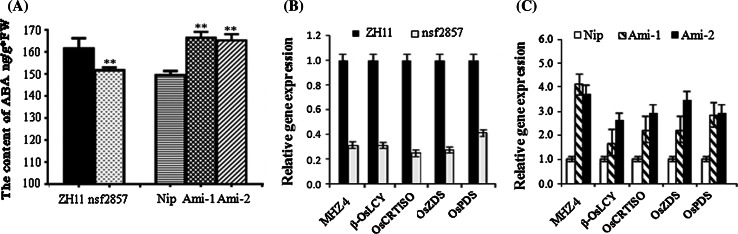
OsERF2 negatively controls ABA accumulation. **a** ABA content of 7-day-old seedlings from ZH11, *nsf2857*, Nip and two *Ami*-*OsERF2* lines. Values represent the means SD of three replicates. **P < 0.01. **b**, **c** Relative expression of ABA biosynthesis genes including *MHZ4*, *β*-*OsLCY*, *OsZDS*, *OsCRTISO* and *OsPDS* in *nsf2857* (**b**), and in two *Ami*-*OsERF2* lines (**c**). *OsACTIN1* was used as an internal control. *Data* represent the means of three repeats

It has been documented that endogenous ABA positively regulates early root growth in rice (Chen et al. 2006), and the basal levels of endogenous ABA are required for the maintenance of normal root elongation in rice (Ma et al. 2014). In this study, we compared the content of ABA in *Ami*-*OsERF2*, *nsf2857* and WT plants. Compared with WT, the content of ABA was decreased in *nsf2857*, but increased in *Ami*-*OsERF2s* (Fig. 3a), suggesting that OsERF2 negatively regulates ABA accumulation. In rice, *MZH4* (*ABA4*) and pre-harvest sprouting genes (*PHS*s), including *OsPDS*, *OsZDS*, *OsCRTISO* and *β*-*OsLCY*, are essential for the synthesis of ABA (Fang et al. 2008; Ma et al. 2014). Here, we found that expression of these genes was downregulated in *nsf2857* (Fig. 3b), but upregulated in *Ami*-*OsERF2* lines (Fig. 3c). These transcriptional changes were consistent with the accumulation of ABA in the corresponding materials.

**Fig. 4:**
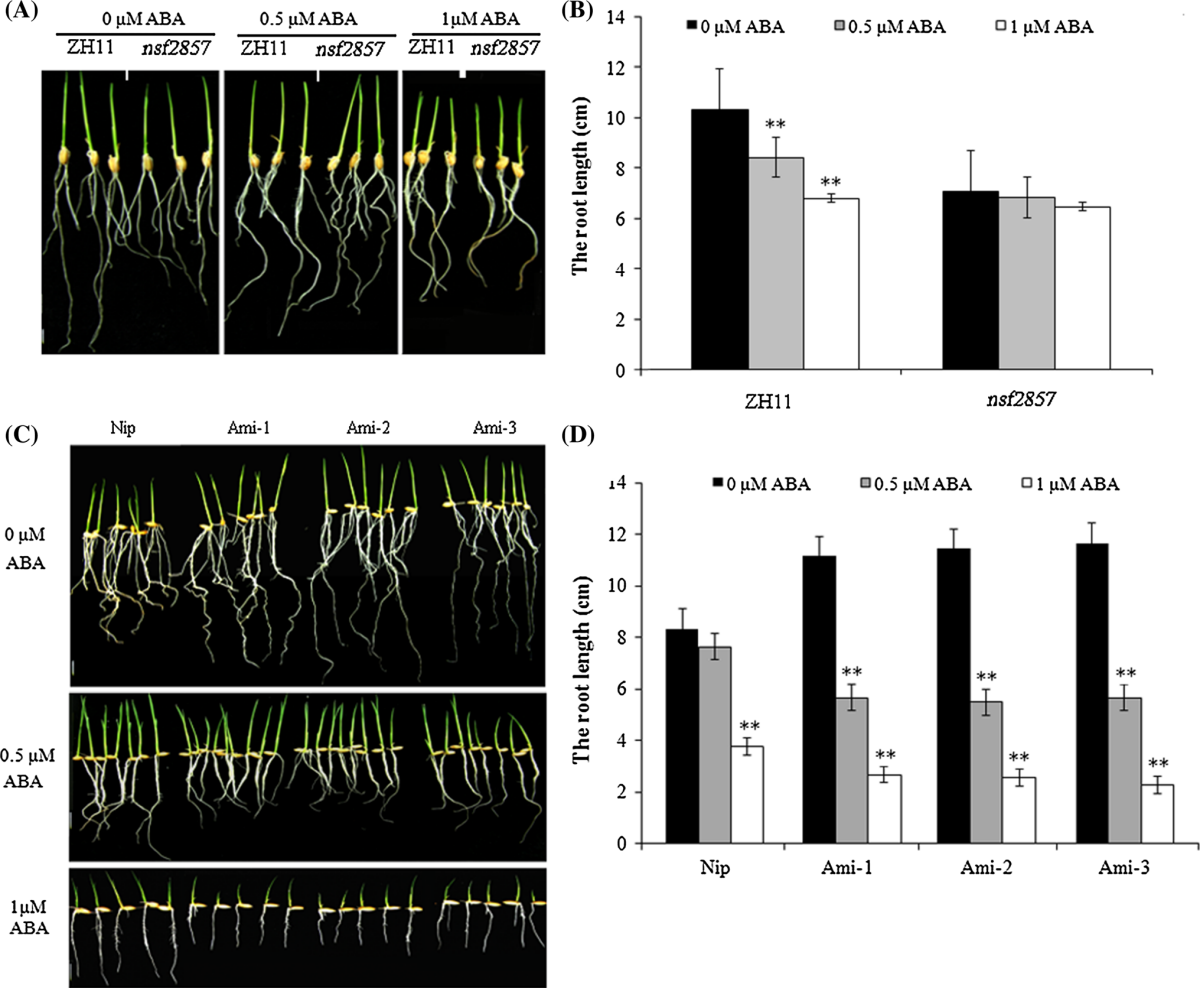
OsERF2 mediates root growth response to ABA. **a** 7-Day-old seedlings of *nsf2857* exhibit insensitivity to ABA compared with ZH11. **c** Seedlings of *Ami*-*OsERF2* lines show hypersensitivity to ABA compared with Nip. *Bar* 1 cm. **b**, **d** Statistical analyses (*t* test) of primary root length of 1-week-old seedlings under 0.5 and 1 μM ABA were performed with graphpad prism 6. *Values* represent the means of three independent biological replicates. **P < 0.01

We then analyzed root growth response to ABA and ethylene treatment in *Ami*-*OsERF2*, *nsf2857* and WT. When treated with 0.5 and 1 μM ABA, the root length of ZH11 decreased from 10.4 to 8.5 and 6.9 cm, respectively, while the root length of nsf2857 was little changed in the presence of ABA (Fig. 4a, b), indicating that upregulation of *OsERF2* caused root insensitivity to ABA in *nsf2857*. In contrast, down regulation of *OsERF2* in *Ami*-*OsERF2* plants resulted in the hypersensitivity of root growth to ABA. As shown in Fig. 3, the average primary root length of three *Ami*-*OsERF2* lines (about 11.41 cm) was longer than that of Nip (8.33 cm) in the absence of ABA. However, the primary root length of *Ami*-*OsERF2* lines decreased to 5.61 and 2.51 cm under the treatment with 0.5 and 1 μM ABA, which were shorter than that of Nip (about 7.63 and 3.78 cm) instead, respectively (Fig. 4c, d). The above results suggest that OsERF2 negatively regulates ABA synthesis and root growth response to ABA, which also confirm that the basal level of endogenous ABA is required for the maintenance of normal root elongation in rice.

**Fig. 5:**
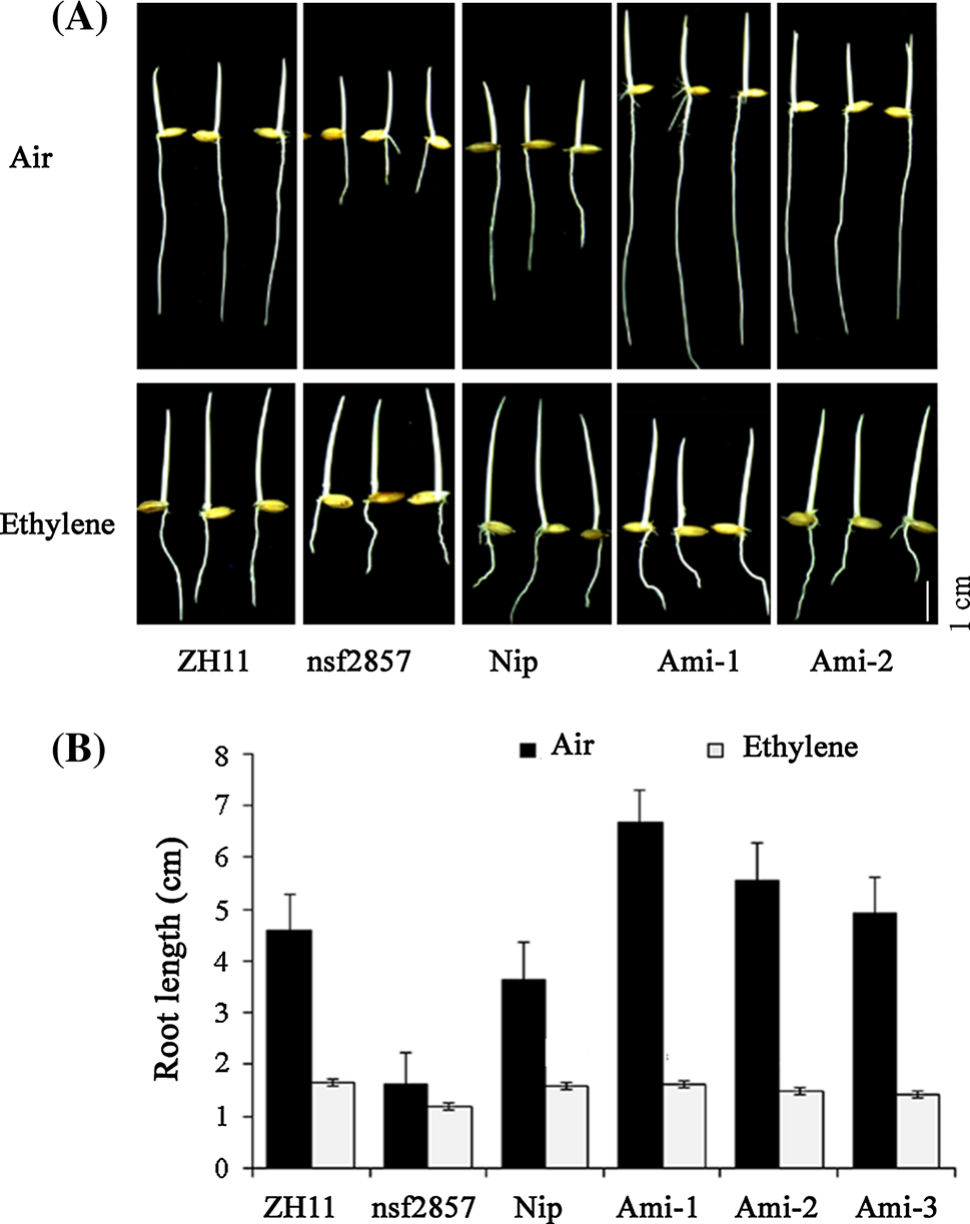
OsERF2 mediates root growth response to ethylene. **a** Ethylene response phenotypes in ZH11, *nsf2857*, Nip and two *Ami*-*OsERF2* lines. Rice seedlings were grown in *dark* for 3 days in the presence or absence of 10 ppm ethylene. *Bar* 1 cm. **b** Statistical analyses (*t* test) of primary root length were performed with graphpad prism 6. *Values* represent the means of three independent biological replicates. **P < 0.01

Upon the ethylene treatment, the primary root growth of ZH11 was significantly inhibited by 64.2 % compared to that in air, but only partially inhibited by 25.7 % in *nsf2857*. Figure 5a, b, indicating that *nsf2857* showed less ethylene-inhibited root growth. In contrast, the root growth of *Ami*-*OsERF2* lines was inhibited by more 71 % compared to that in air, and significantly higher than that of Nip (56 %) (Fig. 5a, b), suggesting that down regulation of *OsERF2* resulted in stronger ethylene-inhibited root growth.

### OsERF2 transcriptionally regulates accumulation of sucrose and UDPG

Sucrose play central roles in the regulation of plant root development by generating a range of signal molecules and interacting with various hormones (Ljung et al. 2015). Here, the contents of sucrose and UDPG (a key precursor for sucrose metabolism) were compared between *nsf2857*, *Ami*-*OsERF2* and WT plants. Compared with corresponding WT, the sucrose of *nsf2857* increased to 112 %, while that of *Ami*-*OsERF2* lines decreased to about 84.7 % (Fig. 5a). In contrast to sucrose, the UDPG of *nsf2857* (22.3 μg/mg) was 88 % of ZH11 (25.1 μg/mg), while that of *Ami*-*OsERF2* (about 26.2 μg/mg) was higher than that of NiP (24.2 μg/mg) (Fig. 5b), suggesting that OsERF2 is involved in regulation of sucrose metabolism. Then, we checked transcripts of several key genes involved in sucrose metabolism including *INV* (Os01g0332100) and *OsCYT*-*INV1* (encoding neutral invertase), *OsSPS2* (encoding sucrose phosphate synthase), *OsSUS3* and *OsSUS6* (encoding sucrose synthase) between *nsf2857*, *Ami*-*OsERF2* and WT plants. As shown in Fig. 5c, d, the expression of candidate genes was upregulated in *Ami*-*OsERF2* plants, but downregulated in *nsf2857*. Particularly, *OsCYT*-*INV1* has been reported to be closely related to the accumulation of sucrose and root growth in rice (Jia et al. 2008). Thus, these results suggest that OsERF2 transcriptionally regulates the accumulation of sucrose and UDPG, which partially contribute to its regulatory roles in rice root growth.

## Discussion

The root system is essential for plants to absorb nutrients and water from soils, which determines plant development progress, response to drought and salinity, as well as crop qualities and yield. Rice is very susceptible to water deficit for its shallow root architecture, but the control of root system can increase its yield under drought conditions (Uga et al. 2013). Recently, many genes and mutants involved in root growth and development have been characterized in rice. Some genes are involved in auxin and cytokinin biosynthesis, transport and homeostasis, and their signal transduction (Azizi et al. 2015; Balzan et al. 2014; Coudert et al. 2010; Mai et al. 2014). For example, the mutation of *CRL5*, encoding an ERF transcription factor, impairs crown root initiation through repression of cytokinin signaling (Kitomi et al. 2011).

**Fig. 6:**
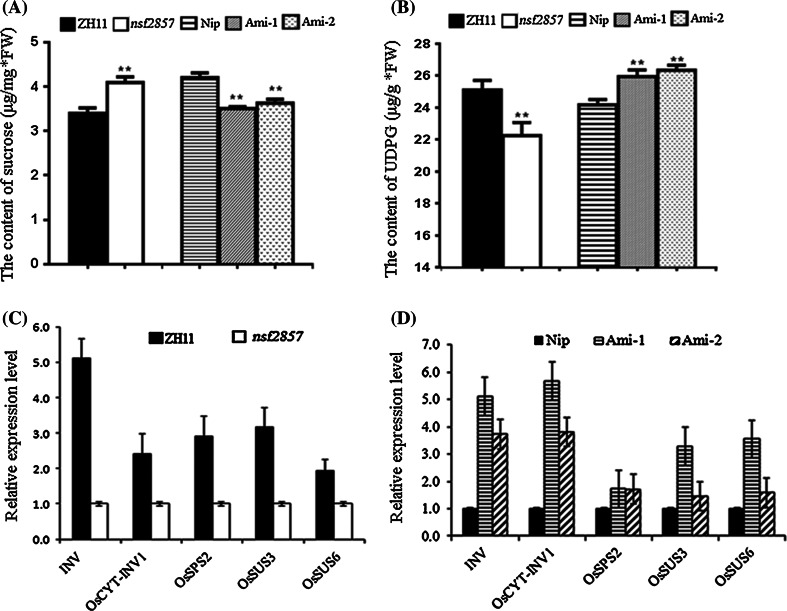
OsERF2 transcriptionally influences the accumulation of sucrose and UDPG. **a** Sucrose content was increased in *nsf2857*, but decreased in *Ami*-*OsERF2* lines. **b** UDPG content was decreased in *nsf2857*, but increased in *Ami*-*OsERF2* lines. Statistical analyses (*t* test) of sucrose and UDPG contents were performed with graphpad prism 6. *Values* represent the means of three replicates. **P < 0.01. **c**, **d** Relative expression of key genes involved in sucrose metabolism in *nsf2857* and *Ami*-*OsERF2* lines. *OsACTIN1* was used as an internal control. *Data* represent the means of three repeats

Although auxin and cytokinin govern plant root architecture, the interplay between ethylene and ABA is required for the control of root growth in rice. For examples, etiolated seedlings of *Oseil1*, *Osein2*, *aba4/mhz4* and *mhz5* exhibited longer primary roots, while their overexpression resulted in shorter roots, respectively. Moreover, the induction of *OsERF2* by ethylene was disrupted in the roots of these mutants (Ma et al. 2013, 2014; Yang et al. 2015; Yin et al. 2015). In the present study, we evidenced that the gain-of-function mutant of *OsERF2* (*nsf2857*) exhibited shorter primary root compared to WT, while down regulation of *OsERF2* by artificial microRNA (*Ami*-*OsERF2*) resulted in longer primary root, suggesting that *OsERF2* has a similar regulatory effect on the primary root growth with *OsEIN2*, *OsEIL1* and *MHZ5*. Thus, we conjecture that OsERF2 might act as a downstream component of ethylene pathway and play key roles in the regulation of root growth (Fig. 6).

ERF family factors play multiple roles in the control of plant metabolism, growth and development, and stress response (Licausi et al. 2013). In rice, many ERFs have been reported to be involved in the regulation of response to various stresses by mediating multiple signaling pathways and expression of stress-related genes (Kazan 2015). Importantly, increasing ERF members are involved in the control of development progress in rice. For examples, OsEATB influences plant height, panicle length and branching by regulating gibberellin biosynthesis (Qi et al. 2011). MFS1, SNB and OsIDS1 control inflorescence architecture and floral meristem establishment through tuning expression of spikelet meristem genes (Lee and An 2012; Ren et al. 2013). Moreover, auxin-induced *CRL5* promotes crown root initiation via repressing cytokinin signaling (Kitomi et al. 2011). In this study, our findings reveal that OsERF2 negatively regulates expression of pivotal genes related to root growth. Among of these candidate genes, *OsMPK3/6*, *OsMKK4/5* and *OsPIN1b/9* are reported to be involved in root growth by tuning auxin and cytokinin signaling (Singh et al. 2015).

ABA affects root growth and development in rice very different from how it does in *Arabidopsis*, and endogenous ABA positively regulates early root growth and is required for the maintenance of root elongation in rice (Chen et al. 2006; Ma et al. 2014). Here, our results suggest that several genes involved in ABA synthesis, including *MZH4*, *OsPDS*, *OsZDS*, *OsCRTISO* and *β*-*OsLCY*, are downregulated in *nsf2857* seedlings, but upregulated in *Ami*-*OsERF2* seedlings. These transcription changes are consistent with the increased ABA accumulation in *Ami*-*OsERF2*, and the decreased ABA level in *nsf2857*. Importantly, it has been proved that *MHZ4* is responsible for ABA biosynthesis and involved in the control of ethylene response, whose mutation and overexpression causes reduced and enhanced ethylene-inhibited root growth, respectively (Ma et al. 2014). In this paper, we found that *Ami*-*OsERF2* lines exhibited longer primary roots and enhanced sensitivity to ABA- and ethylene-treatment, while these phenotypes were the opposite in *nsf2857*. Moreover, our results showed that upregulation of *OsERF2* caused the decreased expression of *MHZ4* and ABA accumulation in *nsf2857*, which displayed similar phenotypes with mhz4 including the shorter roots and reduced ABA- and ethylene-responses. However, Ami-*OsERF2* plants exhibited similar phenotypes with transgenic rice overexpressing *MHZ4*. Taken together, we speculate that OsERF2 is required for the interplay between ethylene and ABA in control of rice root growth, which might be partially related with its transcriptional control of *MHZ4* expression and ABA accumulation.

As the main assimilated carbon of photosynthesis, sucrose play central roles in plant growth and development, as well as stress response by generating series of sugars as metabolites or as signaling molecules interacting the cross talks of metabolic, hormonal, and stress signals (Ljung et al. 2015; Ruan 2014). In *Arabidopsis*, sucrose inhibits hypocotyl elongation and promotes root growth by tuning auxin signal and cotyledon-derived long-distance signal (Kircher and Schopfer 2012; Stokes et al. 2013). In rice, mutation of *OsSPS1*, *OsSUT1*, *OsSUT2*, and *OscFBP1* causes growth retardation and abnormal pollen development by affecting sucrose synthesis or transporter (Eom et al. 2011; Hirose et al. 2010, 2014; Lee et al. 2008). Similarly, UDP-glucose (UDPG) is a key metabolite for the synthesis of sucrose, polysaccharides, glycoproteins, glycolipids and myriads of glycosylated secondary metabolites (Decker et al. 2012). UGPase plays important roles in carbohydrate metabolism through catalyzing the reversible conversion of glucose-1-phosphate to UDPG, which is essential for plant growth, pollen development, and male sterility in rice and *Arabidopsis* (Chen et al. 2007; Park et al. 2010; Woo et al. 2008). In rice, expression levels of genes involved in sucrose metabolism are correlated with sucrose accumulation (Maruyama et al. 2014). In this paper, we found that upregulation of *OsERF2* resulted in the decreased sucrose and the increased UDPG in *nsf2857* plants, while it was the opposite in *Ami*-*OsERF2* plants. Consistent with these physiological changes, expression of key genes closely related to sucrose metabolism including *INV* and *OsCYT*-*INV1*, *OsSPS2*, *OsSUS3*, and *OsSUS6*, were downregulated in *nsf2857*, but upregulated in *Ami*-*OsERF2* lines. Particularly, mutation of *OsCYT*-*INV1* has been proved to cause the enhanced sucrose level and the reduced root growth in rice (Jia et al. 2008). Consistent with this result, we found that *OsCYT*-*INV1* was downregulated in *nsf2857*, which exhibited short primary root and increased accumulation of sucrose. Thus, these findings suggest that OsERF2 transcriptionally controls the accumulation of sucrose and UDPG, which might further trigger the complex alteration of carbohydrate metabolism and multiple signaling pathways, and partially account for the effect of OsERF2 on rice root growth.

## Materials and methods

### Rice materials and treatments

In this study, rice (*O. sativa* L. subsp. *japonica* cv Zhonghua 11 (ZH11) and *O. sativa* L. subsp. *japonica* cv nipponbare (Nip) were used as the wild-type (WT) for *nsf2857* and *amiERF2*, respectively. For root growth and ABA response, more 40 geminated seeds from all samples were planted in 1/2 Murashige and Skoog (MS) medium solution supplemented with 0, 0.5 and 1 μM ABA. After growth for 7 days, root lengths were measured and photographed. For measurement of ABA, sucrose and UDPG, geminated seeds from all samples were planted in 1/2 Murashige and Skoog (MS) medium solution for 2 weeks. All data are presented as means of three times. For ethylene treatment, rice seedlings were grown on a stainless steel sieve placed in an air-tight plastic box with 10 ppm ethylene and in air as control. The seedlings were incubated at 28 °C in the dark for 3 days.

Artificial microRNA (amiRNA-*OsERF2*) construct was generated using specific primer sets (Table S1) designed through programs available on the (http://wmd3.weigelworld.org/), and cloned into the binary vectors pCAMBIA5300. amiRNA-*OsERF2* plants were generated by *Agrobacterium*-mediated transformation and examined by qRT-PCR with specific primers (Table S1). The seeds of *oseil1* mutant and *OsEIL1*-OX were obtained from Dr. Jin-song Zhang.

### Measurements of ABA, sucrose and UDPG

ABA extraction was according to Zhu et al. (2010) measured using the ELISA kit (CSB-E09159Pl, China Agricultural University, China). 0.2 g leaves of 2-week-old seedlings were homogenized and extracted overnight at 4 °C in 80 % methanol by vortex spinning. After centrifuged and concentrated to approximately 50 μl, these extracts were suspended with 1 ml TBS buffer (25 mM Tris–HCl pH 7.5, 100 mM NaCl, 1 mM MgCl2, and 3 mM NaN3) and used for ELISA according to the manufacturer’s instructions. ABA concentrations were calculated as ng/g fresh weight. Each measurement was replicated three times.

Sucrose and UDPG content was measured by HPLC according to Bahaji et al. (2015). 0.1 g 2-week-old seedlings were ground in liquid nitrogen. For measurement of sucrose, the powder was suspended in 1 ml of 90 % ethanol, left at 70 °C for 90 min and centrifuged at 13,000×*g* for 10 min. For measurement of UDPG, the powder was suspended in 1 ml of 1 M HClO_4_, left at 4 °C for 2 h and centrifuged at 13,000×*g* for 10 min. The supernatant was neutralized with K_2_CO_3_ and centrifuged at 13,000×*g* for 10 min. Sucrose was determined by HPLC with UPLC WATERS Acquity system by gradient separation with BEH Amide 2.1 × 100 mm 1.7 μm. UDPG was measured by HPLC on a system obtained from Waters Associates fitted with ZORBAX Carbohydrate 4.6 × 250 mm 5 μm. Standard sucrose and UDPG were purchased from Sigma.

### Gene expression analysis

After geminated seeds were planted in 1/2 MS medium solution for 7 days, total RNA of roots or leaves was isolated from all samples using TRIzol reagent (Invitrogen, Beijing, China) and used to synthesize first-strand cDNA with All-in-One™ First-Strand cDNA Synthesis Kit (Transgen, China). RT-qPCR was performed using gene-specific primers (Table S1) with TransStart Green qPCR SuperMix (Transgen, China). *OsActin1* was used as the internal control. All experiments were repeated three times, and the average was calculated.

## Electronic supplementary material

Supplementary material 1 (DOCX 254 kb)
