# Supplementary material for: OsERF2 controls rice root growth and hormone responses through tuning expression of key genes involved in hormone signaling and sucrose metabolism
Source: Plant Mol Biol. 2015 Dec 10;90:293–302. doi: 10.1007/s11103-015-0416-9 (PMC4717165; doi:10.1007/s11103-015-0416-9)
Supplement: Supplementary file 1 — Supplementary material 1 (DOCX 254 kb) [file 11103_2015_416_MOESM1_ESM.docx]

**Supplementary materials**

Supplementary Figure 1. Check expression level of *OsERF2* in *nsf2857* and *Ami-OsERF2* lines by qRT-PCR. Expression levels of *OsERF2* in *nsf2857* and Nip were standardized as 1. *OsACTIN1* was used as an internal control. Data represent the means of three repeats.


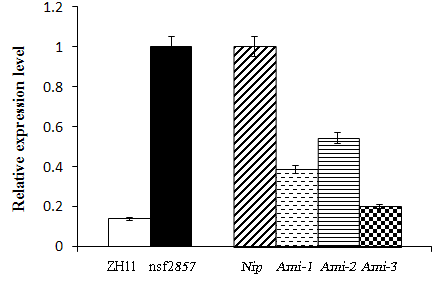


Supplementary Figure 2. Negative effect of OsEIL1 on primary root growth of 7-day-old seedlings. Compared with WT, mutation of *OsEIL1* (os*eil1*) caused the longer primary roots, while transgenic plants overexpressing *OsEIL1* (Os*EIL1-OX*) exhibited shorter primary roots.


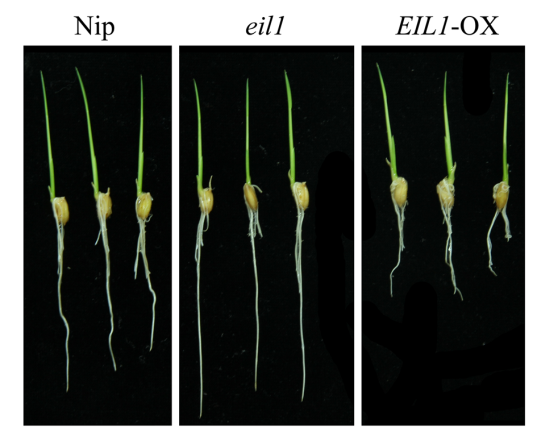


Supplementary table 1. Primers used in this study

| Gene and its ID | Primers |
| --- | --- |
| Gene-speciﬁc primer sets for amiRNA-OsERF2 | |
| I miR-s1th agTTTATGCTCTAAATGATGCTGcaggagattcagtttga,  II miR-a1th tgCAGCATCATTTAGAGCATAAActgctgctgctacagcc,  III miR*s1th ctCAGCAACATATAGAGCATAAAttcctgctgctaggctg,  IV miR*a1th aaTTTATGCTCTATATGTTGCTGagagaggcaaaagtgaa | |
| Primers for qRT-PCR | |
| *OsERF2* Os06g0181700  *OsACTIN1* Os03g0718100  *β-OsLCY* Os02g0190600    *OsZDS* Os07g0204900    OsCRTISO Os11g0572700    *OsPDS* Os03g0184000    *MHZ4* Os01g0128300    *OsCYT-INV1 1*  Os02g0550600    *OsSUS3*  Os07g0616800  *OsSUS6* Os02g0831500  *INV*  Os01g0332100  *KASI* Os06g0196600  *OsMPK3* Os03g0285800    *OsMPK6* Os06g0154500    *OsMPK20-4* Os01g0665200    *OsMKK4* Os02g0787300    *OsMKK5* Os06g0191300    *OsPIN1b* Os02g0743400    *OsPIN2* Os06g0660200    *OsPIN9* Os01g0802700    *ARF1* Os01g0236300  *RHL* Os06g0730200    *OsSPS2* Os01g0919400 | GTGGACCAGATGATCGAGGAG  CCAGAACTCACTGTGACCAA  GGACCCAAGAATGCTAAGCC  TGGTACCCTCATCAGGCATC'  CTGTCGTCGAGGCTCTTCTT  TGGATGAGGTTGCCGATCAT  TGACACATGCTGGAGTGAGT  TCGTGTCAAGGCAAAAAGGG  AGAAAAAGGAGCTTGTGGCG  GGTGTGTTTTTGGTGAGCCT  ACCTCTGCAAAGATCACCGA CTGAGCACAAAGCTTCCCAG  ACCTATCCTGGACTCCCGAC  CGTCATGGTACACCTGCCTT  AATGGCGTATTGTGACAGGC  AGTACTCTGGCCAACCATCC  CGTGTCTGGCTACCACATTG GAAACCGTAGAC ACCGGAG  GGACTCCAACGCATCTATGA  GGCACATTCTTTGCCAACTT  ACAAGCTGAGGGAATACTTAG  GACTCTGCTGTTTTACTCGACG  GCAGAGGTGAACGCCATTAG  AAATTCAACAGCTGGCTCCG  GCGAGGAAGTACATGAGGCA  ATTCTCTGCAGCGGGTTGAA  CGCCAACTTCCTAGACATGC  AGCAGACTGGCTCATCACTT  GCAGACAGAGCTGCCTTACA  AAGTGGCCATTGCACCCTAA  TGTGCTGGCTTCTTGGATTC  TCCCCTCAAAACCACCAGAG  TTCAGAAGCTTCGTCGGCTA  GATGACCGGATGGAATTGGC  CAAGCTTCAACCACACCGAC  GGTACTTGGGCTTGGACGC  GTTCATGGCTCTGCAACCAA  CAATGCCTTGTGGAAGTGCT  TTTCTGATAGGCCCGGTTGT  CCTGTGCTCATGATGTCTGC  GCGTCTTGTGCAGAAACAGA  TCCAACCTTACTAGCCGCAA  ATACTGGTAGGATGAGGCGG  GGATCCTGTGGTGGTTTCCT  ATTTCTGCACGGAGGAAGGA  TCATGGGAGTGTCTTGCTGT |
